# Neuro-interventional Radiology in Paediatric Patients: An Update

**DOI:** 10.1007/s00270-025-04300-7

**Published:** 2025-12-28

**Authors:** Kin Fen Kevin Fung, Moritz Wildgruber, Adam Rennie, Prakash Muthusami

**Affiliations:** 1 Department of Radiology, Hong Kong Children’s Hospital, Kowloon, Hong Kong; 2 Department of Diagnostic and Interventional Radiology, The Hospital for Sick Children, Toronto, Canada; 3 Department of Radiology, University Hospital Munich, LMU Munich, Munich, Germany; 4 Department of Radiology, Great Ormond Street Hospital, London, UK

**Keywords:** Paediatric, Cerebrovascular disorders, Ischaemic stroke, Intracranial arteriovenous malformations, Intracranial aneurysm, Intracranial hypertension, Vein of Galen malformations, Retinoblastoma

## Abstract

Paediatric interventional neuroradiologists play a central role in the multidisciplinary management of cerebrovascular diseases in children. With increasing awareness of endovascular treatment options for paediatric neurovascular diseases and continuous improvement in device technologies, such management is being offered to an increasing cohort of children across the spectrum of diseases and ages. This review article aims to highlight recent advances in neurointerventions in the paediatric population, particularly in the management of arterial ischaemic stroke, brain arteriovenous malformations, vein of Galen malformation, intracranial aneurysms, idiopathic intracranial hypertension, and retinoblastoma.

**Graphical Abstract:**

**Figure:**
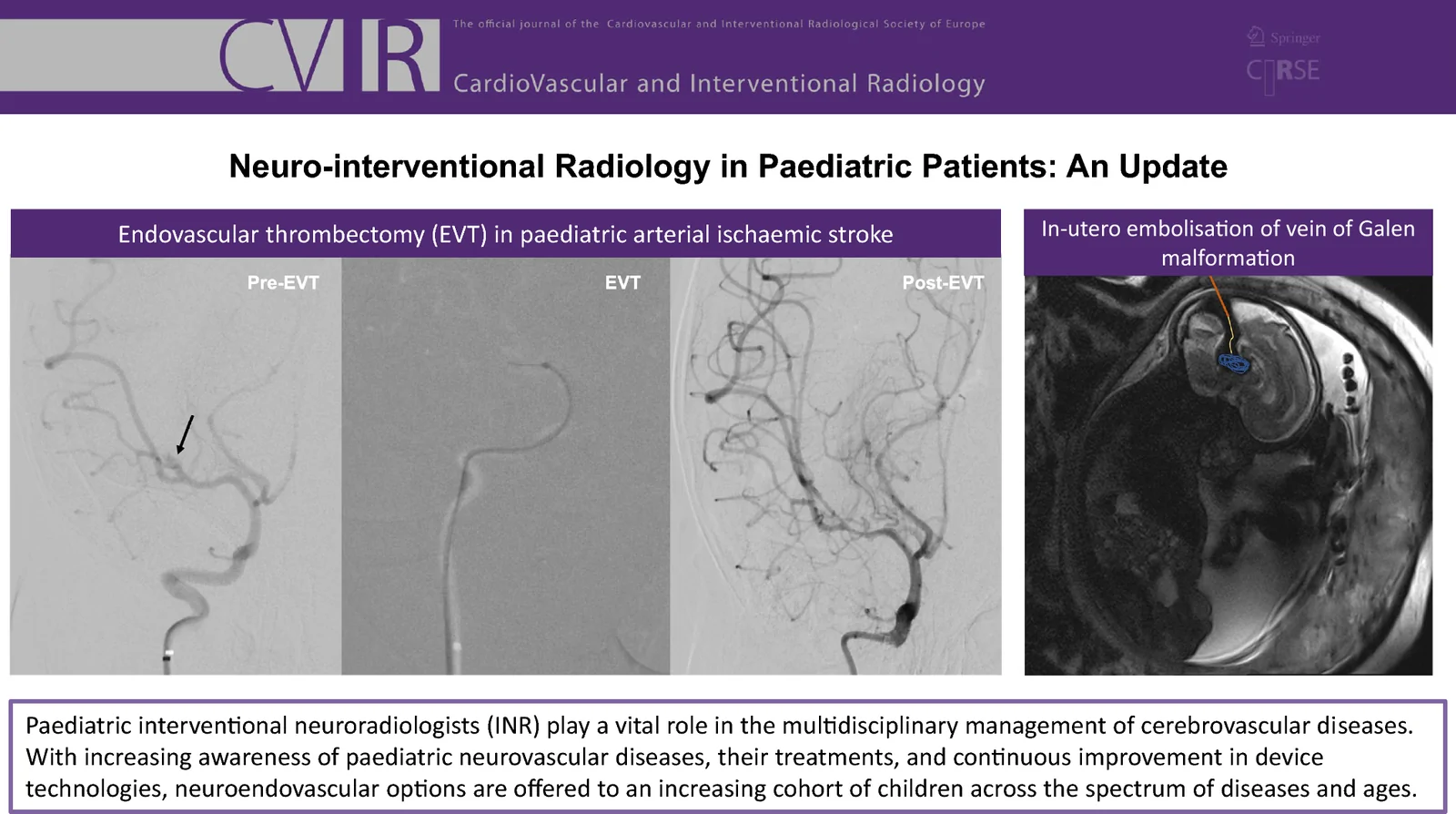

## Introduction

Paediatric interventional neuroradiologists (INR) play a vital role in the multidisciplinary management of cerebrovascular diseases. With increasing awareness of paediatric neurovascular diseases, their treatments, and continuous improvement in device technologies, neuroendovascular options are offered to an increasing cohort of children across the spectrum of diseases and ages. Paediatric expertise of the treating teams is crucial to pre-empt and address specific issues in the care of infants and small children. Paediatric arteries are smaller in size and more prone to vasospasm and dissection. Due to the lack of paediatric-specific equipment, the size mismatch between small arteries and adult-sized vascular sheath/catheter can result in arterial vasospasm, occlusion, and thrombotic complications. Use of US guidance for access, low-profile 4 French hydrophilic radial sheath, prophylactic vasodilator, and systemic heparinisation help mitigate adverse events [1]. Details of these are beyond the scope of this review, which aims to discuss recent advances of paediatric neurointerventions.

## Endovascular Thrombectomy in Childhood Stroke

Arterial ischaemic stroke (AIS) affects about 1.3 – 1.6 per 100,000 children per year in high-income countries. The incidence is estimated to be higher for low- and middle- income countries, especially in areas with a high prevalence of sickle-cell disease [2, 3]. Clinical outcomes of childhood stroke are severe, with 70% retaining long-term neurological deficits, 20% suffering recurrent strokes, and a 10% mortality rate [4–7]. The aetiologies of childhood-onset stroke differ substantially from adults, with the most common causes being focal cerebral arteriopathy (FCA), Moyamoya disease, and cardioembolism [8]. With a large proportion of stroke mimics, the overwhelming positive results from adult endovascular thrombectomy (EVT) trials for large vessel occlusion (LVO) cannot be extrapolated to the paediatric population. The selection of paediatric patients for EVT should be performed in a multidisciplinary setting, led by neurologists familiar with the nuances of paediatric stroke. Similarly, stroke imaging and its interpretation should be performed by paediatric neuroradiologists. These are two—albeit important—parts of a much larger institutional pathway that is required to be established for the efficient, safe, and appropriate use of EVT in children [9].

The retrospective Save ChildS study was the first multicentre study evaluating the safety and effectiveness of EVT in children, demonstrating positive outcomes comparable to those seen in large-scale randomised adult EVT trials [10]. To overcome the lack of a control group in the Save ChildS study, the prospective Save ChildS Pro registry was designed to recruit children with AIS, who were treated either with EVT or best medical treatment (BMT), including systemic thrombolysis, antiplatelet, and anticoagulation [11]. Initial results from the registry confirm the benefit of EVT in children with LVO stroke over BMT [12]. In the EVT group, there was a preference to perform EVT in children who are more severely affected. Despite this, children who received EVT had improved functional outcomes as compared to those who received BMT. Superior outcomes of EVT were demonstrated for both cardioembolic aetiologies and FCA. Caution should be applied when classic features of FCA are present on angiography, such as arterial banding and narrowing of the terminal segment of the internal carotid artery. The optimal selection criteria for patients suitable for EVT, including disease severity at admission and time window for intervention, remain to be answered. Larger prospective studies are required to determine the factors influencing the outcomes of EVT in paediatric LVO stroke and to establish core and penumbral thresholds for EVT in children. [13].

**Fig. 1:**
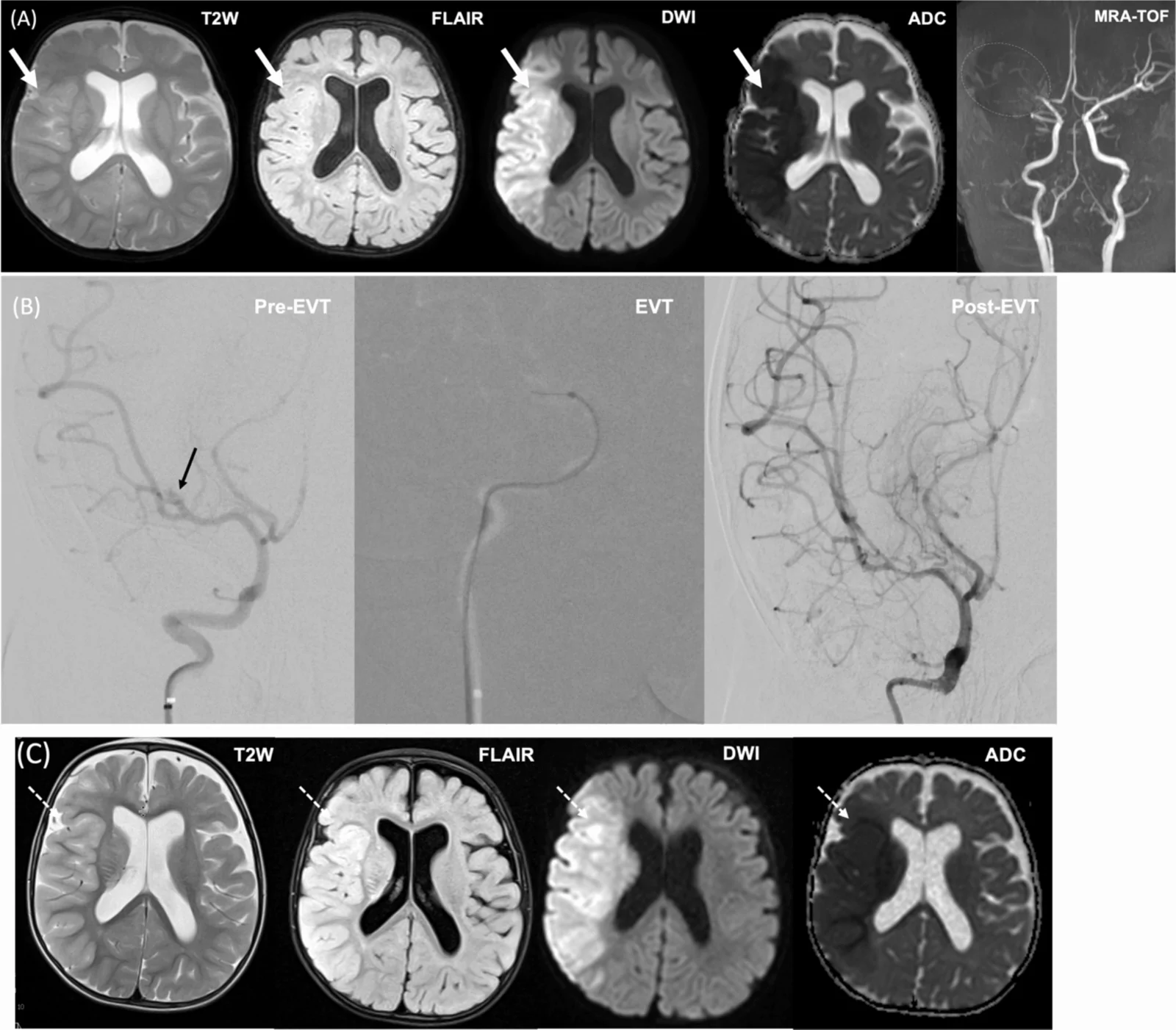
Endovascular thrombectomy (EVT) using aspiration technique in a cardioembolic stroke in a 5-year-old girl with Trisomy 21, who underwent a pericardial patch repair of an atrioventricular septal defect, presenting with acute hemiparesis on the left side. **a** Magnetic resonance (MR) images prior to EVT, at 2 h after onset of symptoms, show acute infarction of right middle cerebral artery (MCA) territory with gryal swelling and restricted diffusion (*white arrow*). Time-of-flight MR angiogram (TOF-MRA) showed absence of right MCA branches (*dashed white circle*). **b** Digital subtraction angiogram (DSA) prior to EVT show truncation of M1 segment of right MCA. After aspiration EVT, the flow to right MCA was successfully restored. **c** MRI 1 day post EVT showed absence of penumbral progression nor haemorrhage in the infarcted region. The patient regained motor function

While EVT was postulated to be risker in children with potential complications such as arterial dissections, vessel rupture, and intracranial haemorrhage (ICH), this trepidation has not been substantiated by the studies published so far. The ICH rates were similarly low in children receiving EVT (8%) or BMT (7%). None of the patients who underwent EVT developed flow-limiting arterial dissections. Transient arterial vasospasm was observed in 15% of the EVT group, but all recovered without clinical sequelae [12]. Adaptation of adult equipment designed for aspiration of distal intracranial vessels may be helpful in infants and young children (Fig. 1). Of note, the youngest patient treated in the Save ChildS Pro registry was 3 months old, and EVT for basilar artery stroke has also been reported in a neonate as young as 14 h after birth [12, 14]. Currently, neither aspiration-based approach nor stent retriever-based approach has shown superiority over each other [15].

## Role for Embolization in Paediatric Brain Arteriovenous Malformations

Brain arteriovenous malformation (bAVM) is the most common cause of spontaneous intracranial haemorrhage in children[16, 17]. bAVMs are mostly sporadic but can be familial in patients with hereditary haemorrhagic telangiectasia (HHT) or RASA-1 mutations[18, 19]. For unruptured bAVMs, the annual risk of rupture ranges from 2% to 3%; and for ruptured bAVMs, the probability of re-rupture in the 1st year can be up to 5% [20, 21]. With a longer life expectancy and neuroplasticity of the paediatric brain, there are robust rationales to advocate for curative therapies for paediatric bAVMs. The goal of nidal bAVM treatment is complete obliteration while preserving normal brain tissue. Depending on the angioarchitecture and haemodynamics of the bAVM, treatment strategies include surgical resection, stereotactic radiotherapy, endovascular embolization, or a combination of these therapies [22, 23].

There is a lack of paediatric-specific guidelines on bAVM treatment. A recent meta-analysis by Pezeshkpour et al., which included 2158 children with bAVMs who underwent treatment or observation, demonstrated that active interventions achieved an obliteration rate of 69.8% (95% CI, 62.9 – 75.9%), and a mortality rate of 2.4% [22]. Most bAVMs treated were Spetzler–Martin (SM) grade 3 or less and nearly 65% of patients presented with intracranial haemorrhage. Long-term outcomes were good, with more than 90% of patients achieving a modified Rankin scale of 0–2. The obliteration and complications rates were 79.4% and 22.5% for surgery, 66.8% and 27.1% for stereotactic radiation, and 54.1% and 26.4% for embolization. Multi-modal treatment was associated with reduced complication rates, which ranged from 5.7 to 12.0%. Mortality and complication rates were 23.5% and 35.9% for conservative management, respectively, which were higher than those of any form of treatment. Based on these results, the authors recommended that curative therapies should be offered to paediatric patients with bAVMs. Multidisciplinary inputs, considering the benefit-risk evaluation, local expertise, and available resources, are essential to formulate individualised treatment decisions [22].

**Fig. 2:**
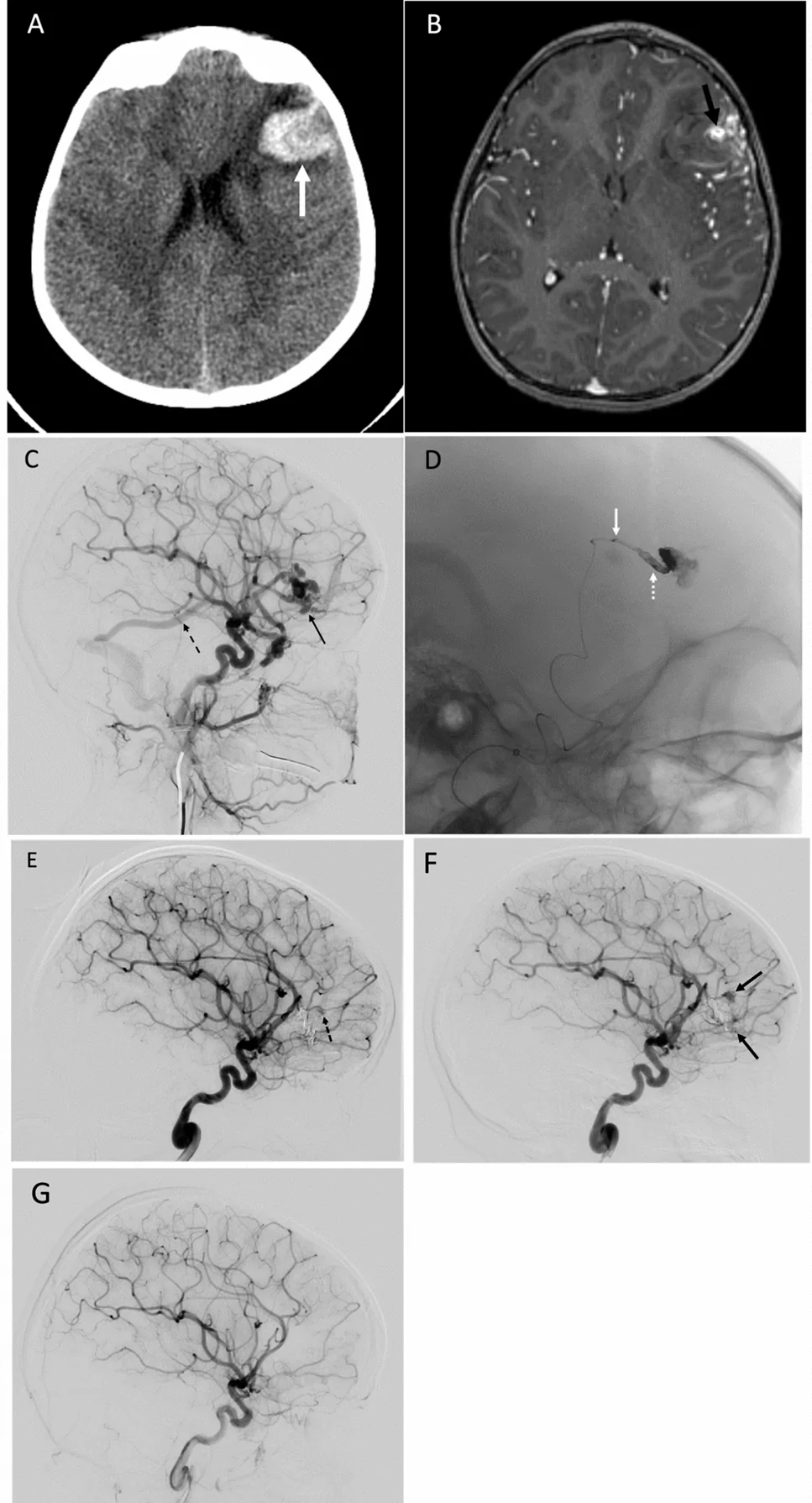
Pre-surgical embolization of left frontal brain arteriovenous malformation (bAVM) in a 5-year-old boy who presented with severe headache. **a** Plain CT brain showing an acute intracerebral haematoma (white arrow) at left frontal lobe. **b** Contrast-enhanced T1-weighed MR image demonstrating a bAVM at left frontal region with a suspicious intra-nidal aneurysm (black arrow). **c** Digital subtraction angiography (DSA) confirmed a left frontal bAVM with early drainage into vein of Labbé (dashed black arrow) and intra-nidal aneurysm (black arrow). **d** Embolization of aneurysm and associated nidus using ethylene–vinyl alcohol copolymer (EVOH) through a microcatheter with detachable tip, such that a plug could be created proximally to allow penetration of EVOH into the aneurysm. Solid white arrow indicates the detachment zone of the microcatheter. Dashed white arrow indicates the distal tip of microcatheter. **e** Follow-up DSA 3-month post-embolization showing persistent occlusion of aneurysm and the residual nidus (dashed arrow). **f** Follow-up DSA 6-month post-embolization showing diffuse nidal enhancement and the child underwent microsurgical resection shortly after. **g** Post-operative DSA showing complete resection of EVOH cast and bAVM nidus

Endovascular embolization plays an increasingly important adjunctive role to improve the nidal obliteration rate and reduce complications of surgery and radiotherapy [24]. In acutely ruptured bAVMs, pre-nidal or intra-nidal aneurysms can be embolized to reduce early re-haemorrhage (Fig. 2) [25, 26]. In one of the authors’ institutions, for unruptured bAVMs, pre-operative trans-arterial embolization is used to decrease overall blood supply to the nidus and has been shown to reduce operative time and intra-operative blood loss [27]. Stereotactic radiation can be used as a surgical alternative for cure in small bAVMs, generally less than 3cm, situated in deep and/or eloquent areas [28]. Embolization can reduce the nidal volume to render patients eligible for stereotactic radiation. Through treatment of high-risk features, including aneurysms and arteriovenous shunts, the risk of post-irradiation haemorrhage can be mitigated [29]. For small bAVMs, single session pre-surgical or pre-radiotherapy embolization is usually sufficient. In larger bAVMs, staged embolization in few weeks’ intervals may be necessary to reduce normal perfusion breakthrough of surrounding oligaemic brain parenchyma [30].

Paediatric bAVMs are associated with a higher rate of recurrence after treatment than adult homologs (Fig. 2E, F), ranging from 4 – 10.7%. An inverse relationship between recurrence and age is observed in the paediatric population. The underlying mechanism is not clearly elucidated but incomplete exclusion due to immaturity of the nidus with continuing de novo cerebral vasculogenesis is believed to play a contributory role.[31].

## Intra-uterine Treatment of Foetal Vein of Galen Malformation

Vein of Galen malformations (VOGMs) are abnormal congenital in-utero shunts between the choroidal arteries and the precursor of vein of Galen, the median prosencephalic vein of Markowski. While this is a rare condition, affecting less than 1 in 25,000 live births, it is associated with significant morbidity, including congestive heart failure, hydrocephalus, and brain parenchymal injury [32, 33]. In modern days, the mortality rate of VOGMs presenting symptomatically in the neonatal period remains high, reported to be around 10 – 15%, and the survivors face high rates of poor neurocognitive outcome [33, 34].

Post-partum embolization remains the standard of care in these patients. While the majority of babies with VOGMs can have their heart failure managed medically and embolization delayed until 3 months, a small cohort of babies who are very sick require immediate embolization to control their heart failure and prevent death. Due to the inherent challenges imposed by the angioarchitecture of VOGMs and neonatal interventions, the rate of intra/post-procedural adverse events can be up to 25%, including cerebral/intraventricular haemorrhage, cerebral ischaemia, hydrocephalus, and vessel perforation.

**Fig. 3:**
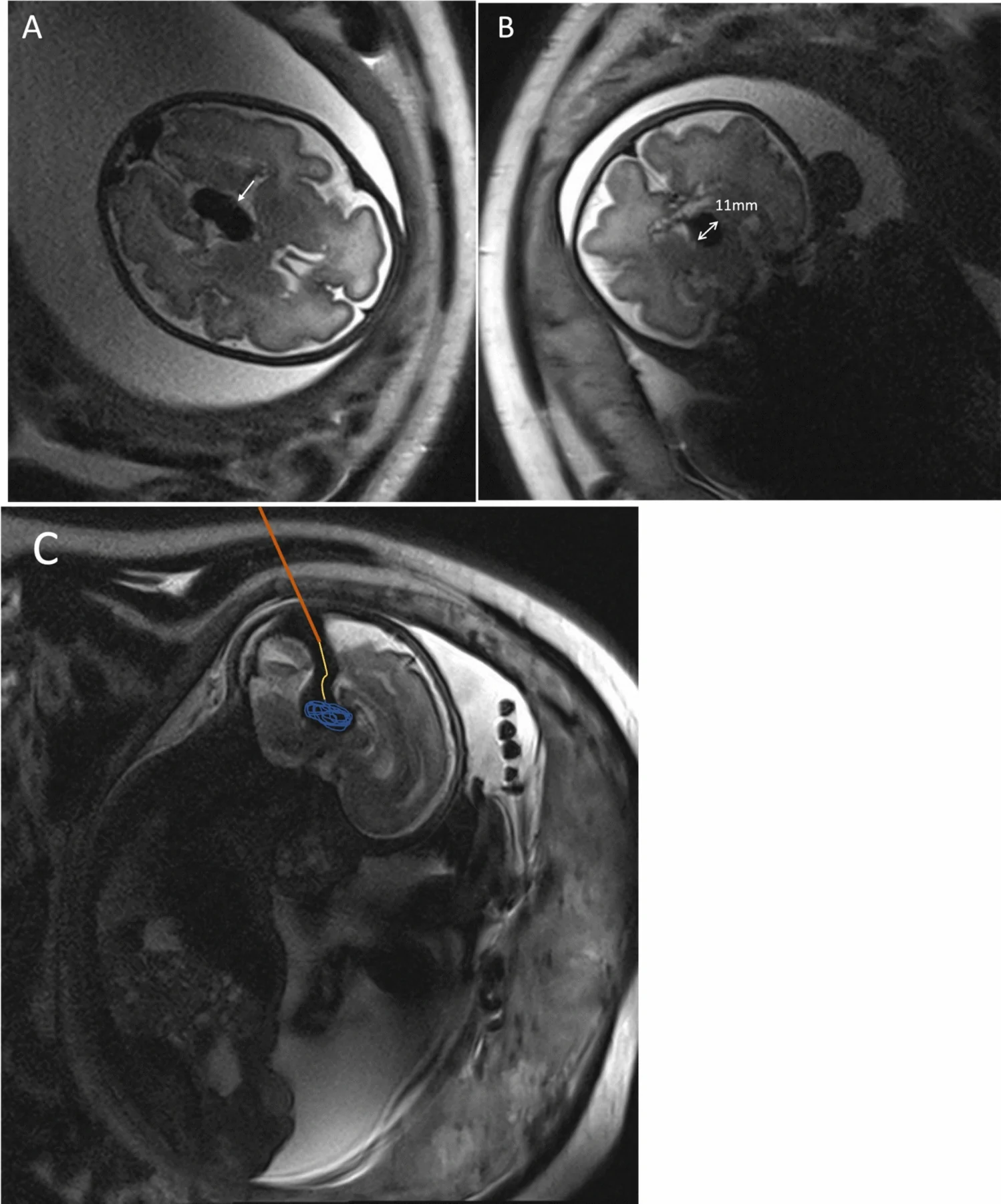
A foetus of 31-week gestational age with vein of Galen malformation. **a** Axial T2-weighed MR showed dilated prosencephalic vein of Markowski (arrow). **b** The medio-lateral width at the falcine sinus constriction measured 11 mm on coronal T2-weighed MR, indicating that there is a high possibility of post-natal decompensation, i.e. neonatal-at-risk population. **c** Illustration showing transuterine coil embolisation of dilated venous varix. A 19G transuterine needle (orange) would be placed into the torcula, through which a microcatheter (yellow) would be navigated to the dilated varix for coil deployment (blue)

Given the protective effect to the foetal heart and brain conferred by the low resistance placental circulation, it has recently been postulated that in-utero embolization of VOGM may decrease morbidity and mortality [35]. The medio-lateral width of the falcine sinus at its tightest point of constriction ≥ 8mm was shown to be a reliable predictor of high-risk VOGMs with acute post-natal decompensation requiring embolization [36] (Fig. 3). A prospective non-controlled trial is currently recruiting pregnant women with foetuses harbouring high-risk VOGMs to evaluate the outcome of transuterine coil embolization of the venous varices of such VOGMs [35]. One of the first successful cases was reported by Orbach et al. on a foetus of 34 weeks and 2 days gestational age, who required no cardiovascular support or post-natal embolization after delivery. MR on post-natal days 1 and 7 demonstrated a reduction in calibre of venous varices and no cerebral parenchymal injury [37]. Following this initial case report, there have been other cases around the world with less predictable outcomes [38–40]. This approach may represent the start of a paradigm shift of VOGM management from a strategy which focuses on reversing the severe multiorgan failure after delivery to a preventative one with in-utero embolization, in a select cohort of neonatal-at-risk babies.

## Reconstructive Strategies for Treatment of Paediatric Brain Aneurysms

Intracranial aneurysms are rare in the paediatric population, constituting less than 5% of all intracranial aneurysms [41]. Paediatric intracranial aneurysms tend to have a fusiform and complex morphology and exist more frequently in the posterior circulation [42]. The incidence of dissecting and fusiform aneurysms is four times higher in the paediatric population, and it was postulated that dissection may be the cause of up to 50% of paediatric intracranial aneurysms [43]. Giant and mirror aneurysms are also disproportionately represented to a greater degree in the paediatric population.

**Fig. 4:**
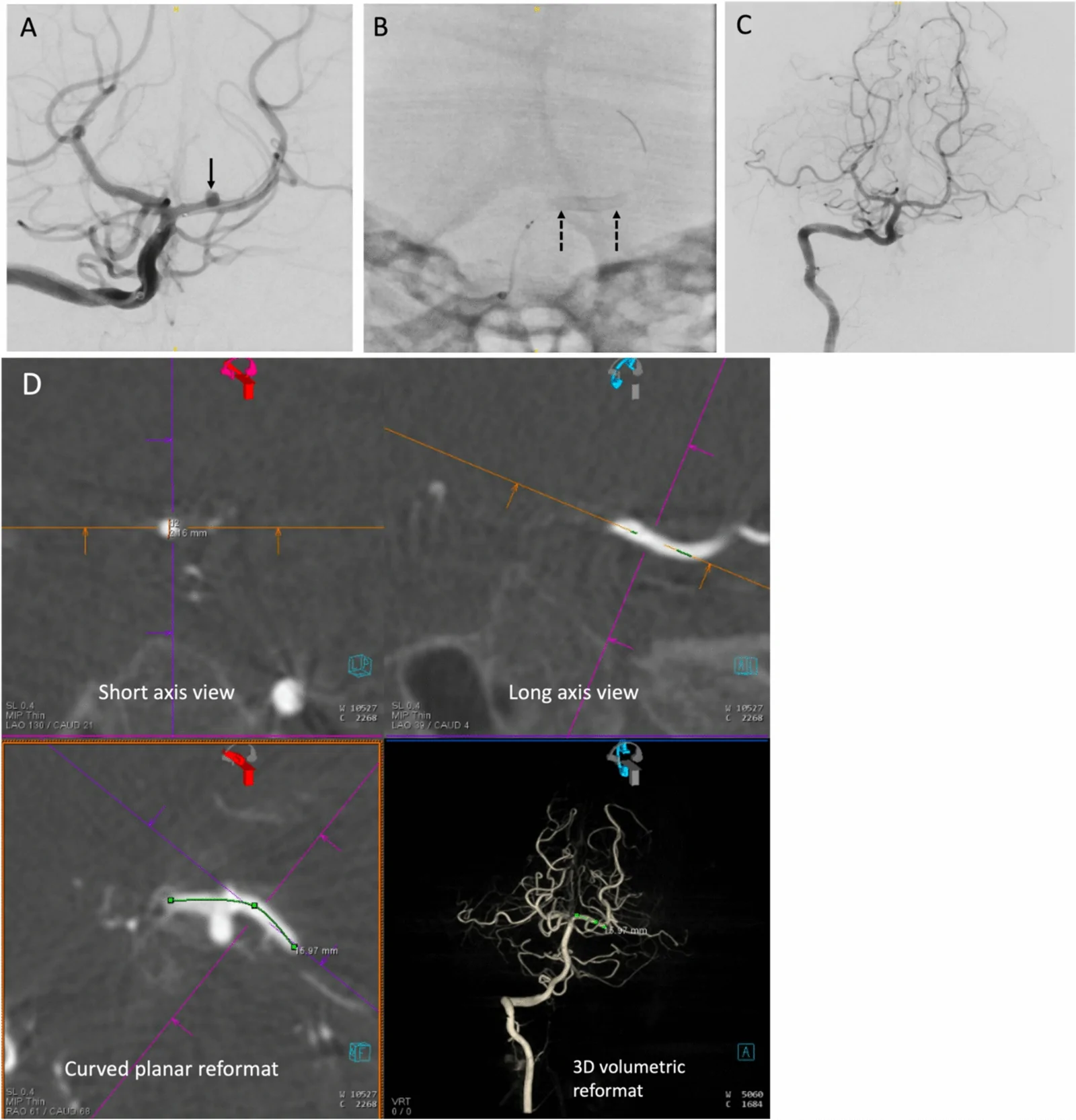
Flow diverter placement in a 7-year-old girl with growing left posterior cerebral artery (PCA) aneurysm. **a** Digital subtraction angiogram (DSA) showing a wide-neck saccular aneurysm (black arrow) at P1 segment of left PCA. **b**. Spot fluoroscopic image confirmed satisfactory position of a 2.25 mm x 15 mm flow diverter stent (dashed black arrow). **c** Post deployment DSA showed preserved left PCA flow and exclusion of left PCA aneurysm. **d** Post-processing of 3D rotational angiogram using multiplanar reformat can help select the appropriate stent diameter and length by measuring the vessel in short axis and curved planar views

Deconstruction with endo-saccular coiling and/or parent artery occlusion remains the mainstay of treating brain aneurysms in children. More recently, flow diverters have been reported as a feasible alternative treatment for brain aneurysms in children when surgical clipping or endovascular coiling can be highly challenging [44–46]. The tightly woven mesh of the flow diverter helps redirect blood flow away from the aneurysm and promotes thrombosis within the sac (Fig. 4). In a recent meta-analysis, which included 80 patients (median age = 9.6 years, range 3–21 years) with 91 aneurysms treated with flow diverters, of which 87.3% were ruptured at presentation, the immediate occlusion rate was 90% (95% CI, 74 – 100%) and the final occlusion rate was 88% (95% CI, 78 – 98%), with a median angiographic follow-up of 10.3 months (range 6 – 22.6 months). The pooled complication rate of 3% (95% CI, 0 – 11%), with the main complication being in-stent thrombosis [45].

The main concerns of flow diverter use in children include the deployment of a fixed-sized stent in growing vessels, size mismatch of relatively large delivery systems in small paediatric vasculature, and use of dual antiplatelet therapy. As most children attain adult-sized intracranial arteries by 4–6 years of life and the cranial arterial diameters do not undergo striking change after early childhood [47], it is unlikely that well apposed flow diverter placement would result in size mismatch with time. Issues with size mismatch between delivery system and femoral artery can be mitigated by use of low-profile hydrophilic radial sheath, which has a thinner wall [48]. The approach to dual antiplatelet medication is extremely variable in the literature, and no published guideline or protocol exists [49, 50]. Consultation with a paediatric haematology/thrombosis team is recommended prior to commencement of such agents. Delayed events which may occur after flow diverter placement include recurrence, change in device configuration, and thromboembolic stroke [44]. While these events are rare and can also be observed in adults, given the longer life expectancy of children, treatment decisions should take these factors into account. Newer intrasaccular devices which are reported in adults have not been adequately tested in children for safety or efficacy.

## Dural Venous Sinus Stenting for Medically Refractory Idiopathic Intracranial Hypertension (IIH)

**Fig. 5:**
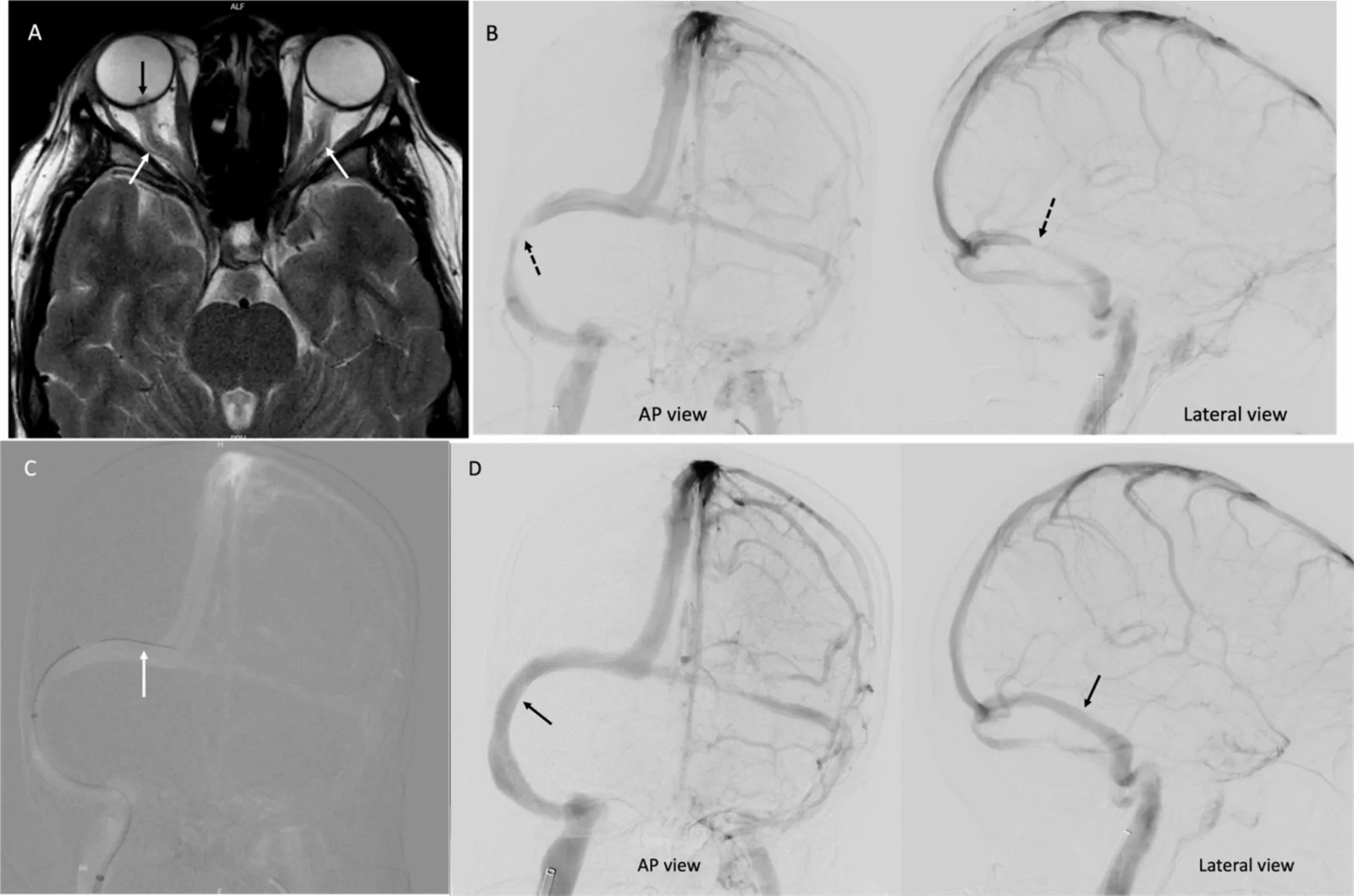
Dural venous sinus stenting in a 15-year-old girl with medically refractory idiopathic intracranial hypertension, who presented with persistent headache and visual disturbance refractory to medical therapy. **a** T2-weighed MR image of orbits showing marked tortuosity of bilateral optic nerves with distended perioptic CSF sheaths (white arrow) and papilloedema (black arrow). **b** AP and lateral projections of digital subtraction venography (DSV) demonstrating severe stenosis at right transverse-sigmoid sinus junction (dashed black arrow). **c** Pressure wire (white arrow) was used to measure the trans-stenotic gradient (TSG), which was 43mmHg. **d** Post-stenting DSV showing significant improvement of stenosis and flow across right transverse-sigmoid sinus junction. The post-stenting TSG was 7mmHg. The headache and visual symptoms improved following dural venous sinus stenting

In the paediatric population, IIH most frequently affects the adolescent age group [51]. Clinically, the elevation of intracranial cerebrospinal fluid (CSF) pressure causes headache, tinnitus, nausea, vomiting, and visual blurring, and if untreated, may progress to visual loss due to papilledema [52]. The pathogenesis of IIH is complex with multifactorial aetiologies, with dural venous sinus stenosis (VSS) being increasingly recognised as a significant contributor [53]. Dural VSS, commonly occurring at the transverse-sigmoid sinus junction, causes a cascade of positive feedback loop as venous hypertension decreases CSF resorption and the resultant increase in CSF pressure exacerbates the anatomic compression on dural venous sinus [52]. Conventional treatment options include lumbar puncture (LP), which has additional diagnostic value (with opening pressure of 25cm H_2_O taken as diagnostic cutoff), and carbonic anhydrase inhibitor, which reduces CSF production. However, in patients who are refractory or intolerant to these treatments and those developing progressive visual loss, dural venous sinus stenting is a minimally invasive solution to preserve vision (Fig. 5) [54].

Despite the small patient cohorts, initial results of dural venous sinus stenting in paediatric IIH patients are promising and echo those in the adult literature [55, 56]. In two small-scale observational studies (8 and 14 patients respectively), most patients had symptomatic improvement (85.7 – 100%) and resolution of papilledema (75 – 100%) post-stenting. A trans-stenotic gradient (TSG) of 8mmHg was taken as the cutoff for treatment under local anaesthesia and 6mmHg for patients under general anaesthesia. The average pre-stenting TSG was 16.1mmHg and the average post-stenting TSG was 1.5mmHg. The rate of re-treatment due to symptomatic recurrence ranges from 25% to 28.6%, with further interventions including re-stenting and CSF diversion surgery. Further prospective and large-scale studies are required to elucidate prognostic factors for post-stenting recurrence.

## Intra-arterial Chemotherapy (IAC) for Retinoblastoma

Retinoblastoma is the most common ocular malignancy in childhood, with an estimated incidence of 1 per 15,000 – 20,000 live births [57, 58, 59]. While the survival rate is high, prior to IAC, almost 80% of patients with advanced retinoblastoma (International Classification Retinoblastoma group [ICRB] group D and E) went on to receive enucleation [60]. The introduction of IAC has significantly improved the globe salvage rate to around 70% – 80% and is considered as the standard of care first-line therapy for advanced unilateral and bilateral disease [57, 60, 61]. In addition, the systemic adverse effect of intravenous chemotherapy (IVC) such as myelosuppression, ototoxicity, and neurotoxicity can be minimised [61]. A recent multicentre randomised trial which compared IAC (72 patients) vs IVC (71 patients) in new-onset unilateral group D or E retinoblastoma without high-risk clinical factors showed that the 2-year progression-free globe salvage rate was significantly higher in the IAC group (53 vs 27%; IAC vs IVC) [62]. Overall survival rates were the same for both groups at 96%.

**Fig. 6:**
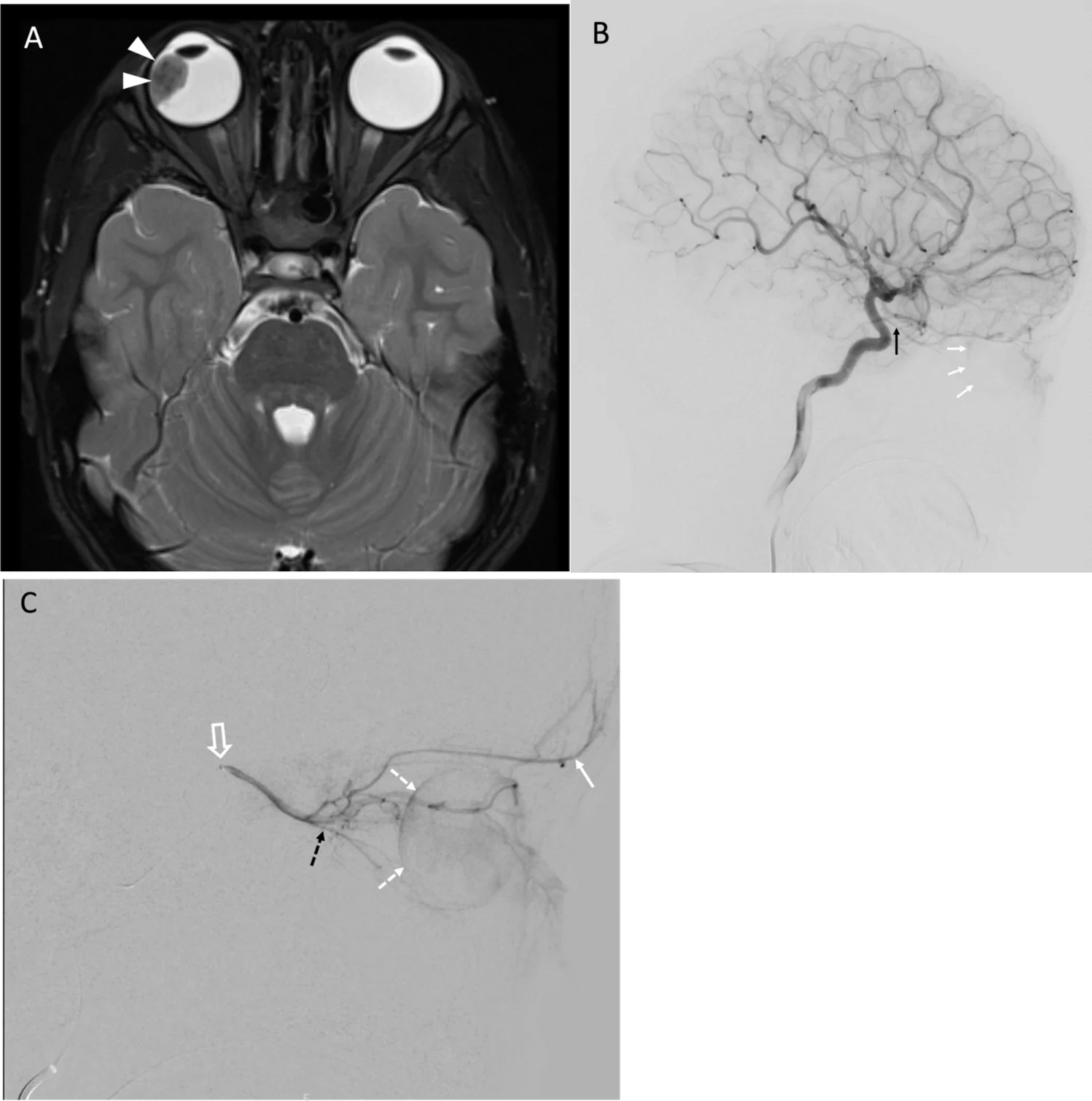
Intra-arterial chemotherapy (IAC) of right eye retinoblastoma in a 2-year-old boy. **a** Axial T2-weighed MRI of orbits showing retinoblastoma involving temporal aspect of right globe. Diffuse vitreous seeding was seen on colour fundoscopy (not shown), in keeping with group D retinoblastoma. **b** Catheter-directed digital subtraction angiography at right internal carotid artery showed conventional origin of right ophthalmic artery (OA, black arrow) with choroidal blush (white arrows). **c** Selective microcatheter injection at right OA ostium (tip indicated by open white arrow) confirmed stable position for IAC administration. Note that the chorioretinal complex (dashed white arrow) is supplied by the central retinal artery and ciliary arteries (dash black arrow). The terminal branch of OA (white arrow) supplies the forehead and scalp region, which explains the occurrence of post-IAC periorbital oedema

The technique of IAC has evolved over the past decades from infusion of chemotherapeutic agents at internal carotid artery (ICA) with distal balloon occlusion to superselective catheterization of the ophthalmic artery (OA) ostium using 1.2 – 1.5 French microcatheters [61]. The reported technical success rate of IAC is very high, reaching up to 98% – 100%, in high volume specialised centres [60]. A good understanding of ophthalmic artery anatomy and determination of dominant arterial supply to the choroid and tumour are essential (Fig. 6). While most choroidal blood supply arises from the ICA and OA, around 10% – 15% patients have preferential supply from ECA via meningo-orbital collaterals or co-dominant supply [63]. Radiation protection measures should be optimised to minimise the risk of secondary cancer development in these radio-sensitive patients [64]. Cardiorespiratory events, including hypotension and bronchospasm, may occur during OA cannulation and were postulated to be due to autonomic reflexes. Recognition of early signs such as decrease in tidal volume and prompt administration of epinephrine can prevent deterioration [65]. Melphalan is the backbone of triple-drug IAC chemotherapy regimen (the other two medications being carboplatin and topotecan); however, it only remains stable for 1 – 2 h after preparation [66]. Therefore, coordination with pharmacy for melphalan preparation and delivery is key to minimise procedural time, prolonged dwell time of microcatheter at OA ostium, and medication wastage [67]. Melphalan can crystalize and embolize ocular arteries causing loss of vision; preventive strategies include filtration through a 2 µm filter prior to infusion, not exceeding the recommended infusion time, and flushing microcatheter with saline after chemoinfusion [68].

Ocular adverse events from IAC are usually mild and self-limiting, including periorbital swelling, ptosis, pupillary changes, forehead rash, retinal detachment, and vitreous haemorrhage [57, 61, 69]. Vision-threatening ophthalmic events, including retinal and choroidal ischaemia, branch or central retinal artery occlusion, or OA occlusion, are rare and reported to be less than 1% in specialised IAC centres [70, 71]. Heparinisation should reduce the embolic stroke risk of the procedure to less than 1%. Systemic chemotherapy toxicity is uncommon and mostly manifests as mild neutropenia. The metastatic rate after IAC is low, ranging from 1.5% to 2.5%, though patients with metastatic recurrence after IAC have significantly poorer prognosis [60, 72].

## Summary

Neuro-interventional radiology continues to evolve, with expanding indications across a diverse range of paediatric cerebrovascular diseases. Interventional neuroradiologists must remain cognizant of the key differences between paediatric and adult patients—including aetiology, pathophysiology, disease spectrum and natural history, clinical presentation, procedural nuances, periprocedural management, and follow-up strategies. The application of emerging techniques and devices requires careful, individualised risk–benefit assessment and close collaboration within a multidisciplinary team to optimise outcome and safety.
